# Providing Personalized Energy Management and Awareness Services for Energy Efficiency in Smart Buildings

**DOI:** 10.3390/s17092054

**Published:** 2017-09-07

**Authors:** Eleni Fotopoulou, Anastasios Zafeiropoulos, Fernando Terroso-Sáenz, Umutcan Şimşek, Aurora González-Vidal, George Tsiolis, Panagiotis Gouvas, Paris Liapis, Anna Fensel, Antonio Skarmeta

**Affiliations:** 1 Ubitech Ltd. Research and Development Department, 15231 Athens, Greece; efotopoulou@ubitech.eu (E.F.); gtsiolis@ubitech.eu (G.T.); pgouvas@ubitech.eu (P.G.); liapis.paris@ubitech.eu (P.L.); 2 Departamento de Ingeniería de la Información y las Comunicaciones, Facultad de Informática, Universidad de Murcia, 30003 Murcia, Spain; fterroso@um.es (F.T.-S.); aurora.gonzalez2@um.es (A.G.-V.); skarmeta@um.es (A.S.); 3 Semantic Technology Institute (STI) Innsbruck, University of Innsbruck, 6020 Innsbruck, Austria; umutcan.simsek@sti2.at (U.S.); anna.fensel@sti2.at (A.F.)

**Keywords:** energy efficiency, behavioral change, personalized recommendations, energy analytics, behavioral analytics, big data analytics, Internet of Things (IoT), Drools, rules management system, semantic reasoning

## Abstract

Considering that the largest part of end-use energy consumption worldwide is associated with the buildings sector, there is an inherent need for the conceptualization, specification, implementation, and instantiation of novel solutions in smart buildings, able to achieve significant reductions in energy consumption through the adoption of energy efficient techniques and the active engagement of the occupants. Towards the design of such solutions, the identification of the main energy consuming factors, trends, and patterns, along with the appropriate modeling and understanding of the occupants’ behavior and the potential for the adoption of environmentally-friendly lifestyle changes have to be realized. In the current article, an innovative energy-aware information technology (IT) ecosystem is presented, aiming to support the design and development of novel personalized energy management and awareness services that can lead to occupants’ behavioral change towards actions that can have a positive impact on energy efficiency. Novel information and communication technologies (ICT) are exploited towards this direction, related mainly to the evolution of the Internet of Things (IoT), data modeling, management and fusion, big data analytics, and personalized recommendation mechanisms. The combination of such technologies has resulted in an open and extensible architectural approach able to exploit in a homogeneous, efficient and scalable way the vast amount of energy, environmental, and behavioral data collected in energy efficiency campaigns and lead to the design of energy management and awareness services targeted to the occupants’ lifestyles. The overall layered architectural approach is detailed, including design and instantiation aspects based on the selection of set of available technologies and tools. Initial results from the usage of the proposed energy aware IT ecosystem in a pilot site at the University of Murcia are presented along with a set of identified open issues for future research.

## 1. Introduction

Energy consumption in residential and commercial buildings is estimated to account for around 40% of total energy consumption, making the need for promoting solutions that can potentially lead to significant reductions compelling. As stated by the U.S. Energy Information Administration, in 2015, about 40% of total U.S. energy consumption was consumed in residential and commercial buildings [1], while a similar percentage is reported by the European Commission for the overall consumption of the buildings sector in the EU [2].

The design and adoption of novel information and communication technologies (ICT) towards achieving higher levels of energy efficiency in the buildings sector is considered promising, as stated in the Global e-Sustainability Initiative SMARTer2030 report [3]. ICT has the potential to enable a 20% reduction of global CO_2_ equivalent emissions by 2030, holding emissions at 2015 levels [3]. The application of ICT-enabled solutions is going to provide residents with greater insight and control, and an enhanced living experience whilst saving energy and resources. However, the application of novel ICT technologies for energy efficiency has also to rely on people adjusting their energy consumption behavior. As stated in the report of European Environment Agency [4], up to 20% of energy savings can be achieved through different measures targeting consumer behavior.

In this article, ENTROPY, an energy-aware IT ecosystem is detailed. ENTROPY aims to support energy efficiency in the buildings sector through behavioral change of the occupants with regards to their daily energy consumption patterns [5]. The main distinguishing characteristic of ENTROPY is that it exploits the advantages provided by a set of novel ICT technologies for enabling the design, development and provision of personalized energy management and awareness services in smart buildings. The philosophy of the proposed approach is based on the provision of personalized services that can lead to behavioral change through energy consumption awareness and motives provided to occupants based on their behavioral profile.

The main adopted ICT technologies include Internet of Things (IoT), information fusion, semantic web, rule-based recommendations, big data mining, and analysis mechanisms. Novel IoT node configuration, networking, and efficient data aggregation mechanisms, including mobile crowd-sensing mechanisms, are applied for the interconnection of any type of sensor (e.g., low-cost sensors such as Arduino and Raspberry Pi), a collection of data, and the application of data quality enhancement mechanisms (e.g., removal of outliers, fix missing values in time-series data). Information processing, semantic mapping, and fusion mechanisms are applied for representing the collected data in a unified way, boosting in this way their exploitability and interoperability with existing services, as well as their interlinking with available data. Recommendation mechanisms are applied for real-time reasoning over the available data and provision of suggestions for personalized actions that can lead to improving energy efficiency through behavioral change. Big data mining and analysis mechanisms are also supported for producing behavioral and energy consumption analytics, targeting at providing advanced insights and increasing the energy-awareness level of end users.

All the aforementioned technologies are supported through an integrated IT ecosystem that comprises the basis for the consumption of existing services, as well as the design and development of further energy management and awareness services, personalized mobile applications, and serious games.

The structure of the paper is as follows: In Section 2, the overall architectural approach for the design of the energy-aware IT ecosystem is provided, including subsections for the description of the IoT nodes management and aggregation mechanisms, the description of the two designed semantic models for representing energy management and occupants’ behavioral concepts, and the description of the set of services provided to end users, namely, the personalized recommendations services, the data mining and analysis services, and the set of APIs for the development of personalized mobile applications and serious games. Following this, in Section 3, initial results based on the deployment of the proposed IT ecosystem at the University of Murcia in Spain and the realization of an energy efficiency campaign are presented, while Section 4 provides a set of conclusions and identifies open issues for future research.

## 2. Energy-Aware IT Ecosystem Architectural Approach

Prior to delving into the description of the ENTROPY energy-aware IT ecosystem architectural approach, the type of users that interact with the ecosystem along with a concise overview of an indicative workflow for realizing an energy efficiency campaign is provided. Two types of users are considered in ENTROPY, namely, campaign managers and end users. Campaign managers are responsible for setting up an energy efficiency campaign and may consist of smart buildings administrators, energy efficiency experts, data scientists and behavioral scientists. The combination of knowledge, with regard to energy efficiency, data science, and behavioral aspects, is considered necessary towards the setup of sensor data monitoring, data analysis, and personalized recommendation delivery processes considering the infrastructure in the smart buildings and the type of users engaged in the campaign. End users regard the set of users participating in the campaign and they may consist of citizens, students, academic personnel, employees in enterprises, etc.

**Figure 1:**
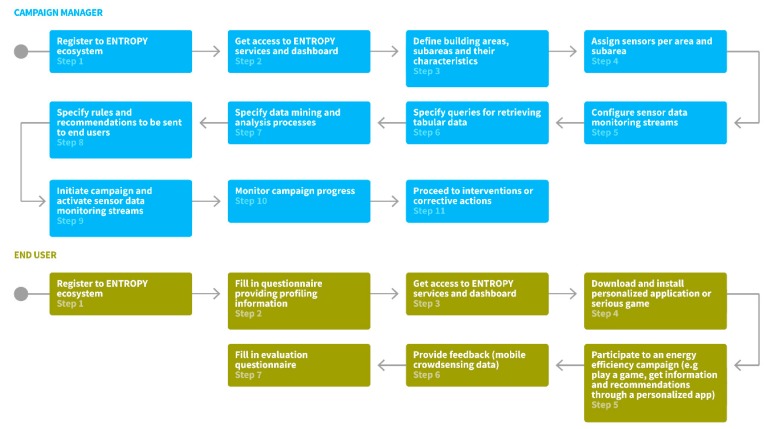
ENTROPY users and basic platform usage workflow.

The basic steps followed by campaign managers for initiating and running an energy efficiency campaign are depicted in Figure 1. Upon registration to the ENTROPY ecosystem, a campaign manager obtains access to the ENTROPY services and is able to define the set of buildings taking place in the campaign, along with their division in subareas. For each area or subarea, a set of characteristics related to the surface, the working hours, the capacity in number of people, the location, the energy class of the building, etc., are provided. The next steps regarding the assignment of sensors per area or subarea and the configuration of the set of sensor data monitoring streams that have to be activated. Based on these, a set of data queries can be designed through a query design editor, the results of which are being used as input for data mining and analysis processes. The latter also have to be specified including information regarding the algorithm to be executed and the type of input and output data. Following this, the campaign can be initiated through the activation of the sensor data monitoring streams and the initiation of interaction with the end users. Continuous monitoring, evaluation and undertaking of corrective actions can be realized by the campaign administrator. It should be noted that the aforementioned steps do not need to be followed in a strict sequential way, depending on the specificities of each campaign.

The basic steps followed by end users for participating at an energy efficiency campaign are also depicted in Figure 1. Upon registration to the ENTROPY ecosystem, the end user has to fill in a questionnaire targeted at providing a user profile with regards to the type of employee personality, work engagement, energy conservation habits, and game interaction preferences. Following, the end user gets access to the ENTROPY services and is able to install and run ENTROPY mobile applications and serious games and, thus, participate to an energy efficiency campaign. Through the ENTROPY applications, the end user gets information regarding energy consumption, as well as environmental parameters in the areas that he has activities, receives personalized recommendations and requests for action while he is also able to provide feedback to the ENTROPY platform (e.g., information regarding malfunctioning of equipment). At the end of an energy efficiency campaign, the end user is requested to fill in an evaluation questionnaire, targeting at measuring perception of behavioral change, as well as any changes with regards to their gaming profile.

**Figure 2:**
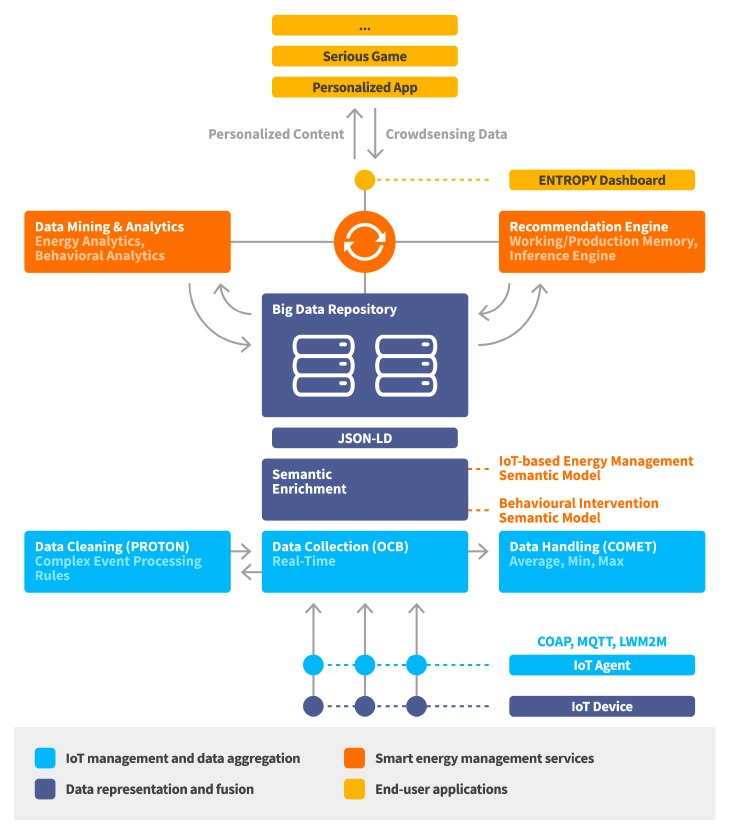
Energy-aware IT ecosystem architectural approach.

A high-level view of the ENTROPY energy-aware IT ecosystem architectural approach is provided at Figure 2. As depicted, a layered architecture is followed with discrete layers for IoT management and data aggregation, data representation and fusion, smart energy management services and end user applications. The IoT management and data aggregation layer is responsible for IoT nodes registration, management and data aggregation and cleaning functionalities at the edge part of the infrastructure. The data representation and fusion layer is responsible for representing the collected data based on a set of defined semantic models as well as supporting a set of data fusion mechanisms over active data streams. The smart energy management services layer is responsible for providing advanced analytics and recommendations to end users, as well as incorporating learning techniques for continuously exploiting the produced output by each service. The end user applications layer is responsible for the design of personalized mobile applications and web-based serious games able to take advantage of the set of services provided by the lower layers. Following this, detailed information is provided for the designed and implemented mechanisms per layer.

### 2.1. Internet of Things Node Management and Data Aggregation

The mechanisms designed for the IoT management and data aggregation layer follow an edge computing approach. Edge computing facilitates the processing of information, where required, in the logical extremes of a network, improving in this way the performance and efficiency of applications in terms of usage of resources. It should be noted that the design of energy and information management systems is considered one of the main application areas that combine IoT and edge computing technologies [6,7]. The set of mechanisms support the easy registration, configuration and lightweight management of the infrastructure sensors deployed in the target buildings and a set of data aggregation, pre-processing and cleaning functionalities. The design and development of such mechanisms is based on the adoption and extension of a set of cloud-based open enablers, provided by the European platform for Future Internet FIWARE [8]. These enablers are orchestrated together by means of lightweight RESTful Application Programming Interfaces (APIs) according to the Open Mobile Alliance Next Generation Service Interface (NGSI) 9–10 standard [9].

In the provided approach, a set of different enablers provided by the FIWARE platform may be used, given that we are based on decoupled and self-contained modules. Data access and processing mechanisms can be designed in a future-proof way, given that the NGSI standard intends to provide a uniform cross-domain interface for advanced data access and processing. Since FIWARE enablers are compliant with such an interface, this facilitates the interoperability of FIWARE solutions with other architectures avoiding a silo-effect and making FIWARE an open solution that can be easily adopted by private and public stakeholders.

It should be noted that a set of initiatives and approaches are using FIWARE enablers in various domains. Indicatively, the Global Services Mobile Alliance (GSMA) has defined a generalized architecture for the delivery of “Internet of Things” “Big Data” services to support an ecosystem of third-party application developers [10]. Within this architectural approach, the FIWARE NGSIv2 interface has been specified as the recommended standard for certain interfaces, while a set of FIWARE enablers are used for supporting specific functionalities, including the data and control broker. In [11], a system architecture for achieving world-wide semantic interoperability solution is presented, combining the NGSI, which is part of the core of the FIWARE initiative, and oneM2M context interfaces. In [12], a semantic mechanism to integrate data from different types of devices by using FIWARE components is presented, while, in a more functional domain, and [13] made use of certain enablers, like the ORION context broker, to create a cloud-based gesture recognition application. Furthermore, in [14] it is described a sensor management system for seaports based on the FIWARE platform, while in [15] a novel patient monitoring system based on FIWARE enablers is proposed. Following the existing works on a set of diverse domains, the proposed work in this manuscript in one of the first efforts to make use of FIWARE enablers in the energy management in smart buildings domain and, thus, in the energy domain in general.

Regarding the usage of the FIWARE enablers in the ENTROPY ecosystem, the first step regards the registration of the sensor nodes and the collection of sensor data in real-time. The FIWARE enabler called IoT Agent is used for this purpose [16]. The IoT Agent acts as a gateway for hardware devices. It supports a set of communication protocols (e.g., Constrained Application Protocol (COAP), MQ Telemetry Transport (MQTT), Lightweight Machine-to-Machine (LWM2M)) for establishing connectivity with the sensor nodes and retrieving data in real-time.

The way that this data is stored and managed is tackled by another FIWARE enabler called Orion Context Broker (OCB) [17]. OCB supports the creation of real or virtual elements of interest by using the term “entities”. Each entity is considered as a virtual sensor node that can obtain data from infrastructure sensor nodes. In the ENTROPY ecosystem, an information model comprising one entity per type of infrastructure sensor has been defined, facilitating the collection of data for all the registered sensor nodes in a homogeneous way. For instance, for collecting energy consumption data, an entity type named “energy_sensor” representing an energy meter installed in the considered building is created. Each entity includes the set of attributes monitored by the infrastructure sensor nodes along with metadata regarding the location of the sensor and the timestamp of each observation.

Following, a sensor data cleaning process takes place for improving the overall data quality through the usage of the FIWARE Complex Event Processing (CEP) enabler called PROTON [18]. CEP focuses on timely processing streams of information items, so-called events, like filtering or aggregation by means of predefined rules following the event-condition-action paradigm [19]. A filtering mechanism is implemented that discards extreme outliers of the different attributes that a sensor measurement contains, avoiding the further transmission of erroneous data. Specifically, a first set of CEP rules focus on calculating certain statistical features of each sensor’s attribute stream (e.g., first, third, and inter-quartile values) over a particular time window. Next, a second set of CEP rules is applied for each new sensor observation, discarding it in case it is considered as an outlier based on the previously defined statistical features.

Given the existence of high quality data, the COMET FIWARE enabler is used for supporting access to historic time series data [20]. COMET adheres to the same information models as the OCB enabler that gets the real-time data, thus, it does not require any further data harmonization process. It incorporates several built-in simple aggregation functions over the historic sensor data (e.g., provide sum, minimum, or maximum values). Access to such data is considered very helpful for realizing comparisons, providing input to data mining and analysis processes as well as describing rules that can lead to personalized recommendations.

### 2.2. Semantic Representation Models and Data Fusion

**Figure 3:**
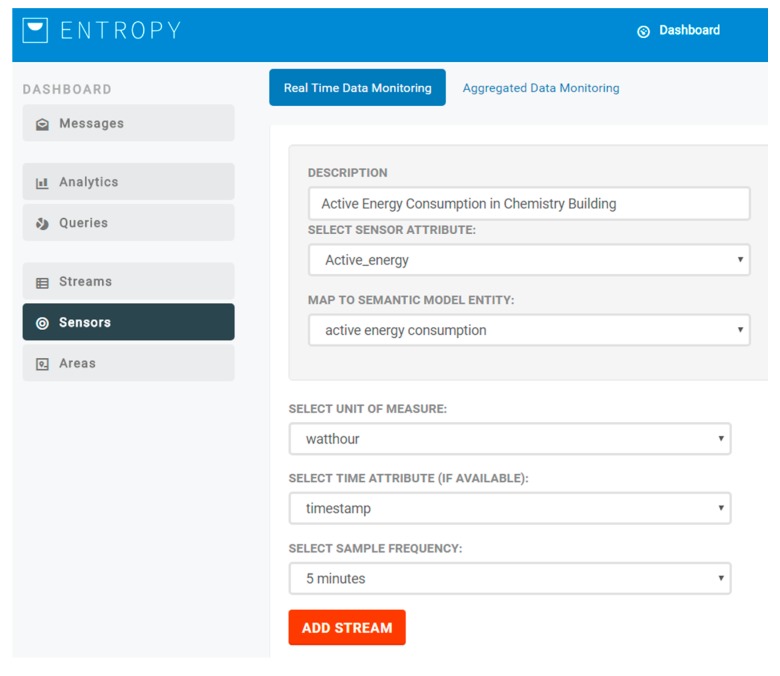
Sensor data stream configuration.

Upon making the collected data available through the IoT management and aggregation layer, sensor data streams towards the ENTROPY platform can be activated. Sensor data streams may regard real-time data or aggregated data. For each of the activated data streams, the collected data is mapped to the ENTROPY semantic models [21,22], as detailed in the following subsections, and then stored in the big data repository that is based on MongoDB. The semantic enrichment module is integrated with the ENTROPY platform and operates as an intermediate layer between data coming from FIWARE and the ENTROPY platform (user interface and REST-API) and the MongoDB. Upon the activation of a new sensor data stream, the end user is responsible to denote the mapping between the monitored sensor metric with the relevant parameter in the semantic model, as depicted in Figure 3. Following this, the collected data is stored based on the semantic model denoted parameter, supporting the unified access to the collected data.

The semantic models provide a set of advantages in terms of management and exploitation of the collected data. Through the representation of the main concepts and their relations and the mapping of the data into these concepts, unified data representation and data access mechanisms are designed and implemented. Exchange and reuse of data are also facilitated, especially following the evolving open and linked data principles. By exploiting the plethora of existing linked data tools, interlinking of data collected via the sensor data streams with available open or privately-owned data can be realized. Within ENTROPY, interconnection with the LinDA workbench [23] is realized, that is a complete open-source package of enterprise linked data tools to quickly map and publish your data in the linked data format, interlink them with other public or private data, analyze them, and create visualizations. Such functionalities for data interconnection, exchange and reuse are considered crucial for energy data in the buildings sector for realizing comparisons among data collected via similar campaigns in different regions, as well as setting up target values for energy efficiency. Interconnection of energy data with other environmental monitoring parameters provided as open data by meteorological authorities or socioeconomic data made available by national or international statistics authorities can be also achieved (e.g., similar to the study realized in [24]), leading to advanced analysis and insights without requiring too much data management effort on behalf of the data scientists. Furthermore, through the semantically-represented data, reasoning mechanisms may be applied by taking advantage of the denoted semantics and leading to advanced insights or recommendations, as they are detailed in Section 2.3.1. Finally, linked data analytics can be produced considering the representation of a data mining and analysis process in the semantic models and the interlinking of the input and output datasets of an analysis. The enriched datasets can be exploited in a twofold way. On one hand, they can be used by data scientists for analysis results comparison purposes, while on the other hand, they can be used for defining set of rules for producing recommendations considering the analysis results.

As already mentioned, data is stored in a MongoDB database that is a NoSQL database. Such a choice is made mainly by taking into account the supported load balancing and sharding characteristics. In order to support the storage of data without losing their expressivity in terms of their mapping to the semantic models and in parallel ensure high-performance characteristics during data management and reasoning processes, data is stored in JSON-LD format that stands for JavaScript Object Notation for Linked Data. JSON-LD is a method of encoding linked data using JSON. It is considered as an ideal data format for programming environments, REST web services, and unstructured databases, such as MongoDB. Using MongoDB and JSON-LD together is considered optimal in cases that combination of efficient representation schemes along with efficient data retrieval mechanisms has to be realized. Actually, JSON-LD was created for developers who are working with data and it showcases the power of linked data without having to go through the somewhat steep learning curve that the semantic web usually has. JSON-LD facilitates publishing data through APIs while it splits the representation layer (HTML) from the semantic layer (JSON-LD), characteristics that are not supported through other formats (e.g., Resource Description Framework—RDF). Finally, the representation of the data in JSON-LD format enables the design and implementation of reasoning mechanisms that overcome the performance limitations of ontology-based reasoning through the processing of SPARQL rules. Such an approach has been adopted in the presented IT ecosystem, through the adoption of Drools for semantic reasoning purposes over JSON-LD-represented data.

#### 2.2.1. IoT-Based Energy Management Semantic Model

**Figure 4:**
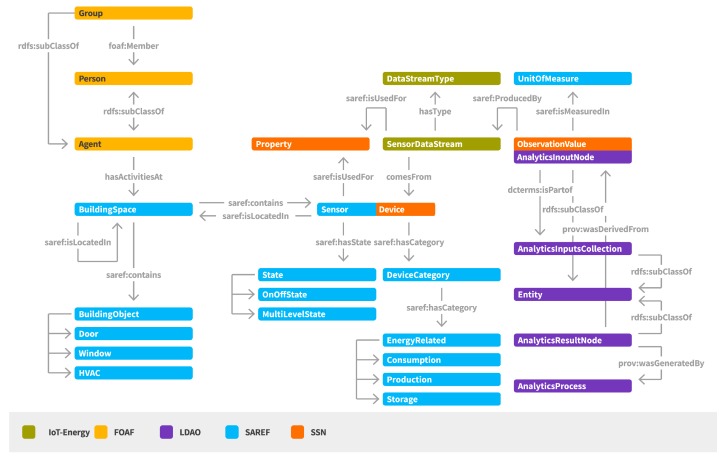
IoT-based energy management semantic model.

The IoT-based Energy Management semantic model (IoT-Energy) [21,22] aims to represent the set of concepts related to the support of energy efficiency in smart buildings (Figure 4). It includes conceptualization of the buildings, their structure, the deployed sensor networking infrastructure, the activation of sets of sensor data streams, as well as the realization of analysis over the collected sensor data. IoT-Energy inherits and builds upon well-known ontologies, such as the Friends of a Friend (FoaF), the Smart Appliances REFerence (SAREF), the Semantic Sensor Network (SSN), and the Linked Data Analytics Ontology (LDAO) [22,23].

A main entity regards the *BuildingSpace* that represents any space in the building. A *BuildingSpace* may be located in another *BuildingSpace*, supporting in this way a hierarchy of building spaces. A set of *Persons* or *Groups* may have activities at a *BuildingSpace*, information that is highly helpful for providing personalized content and recommendations to the associated persons per location.

A *BuildingSpace* contains *BuildingObjects* and *Sensors/Devices*. By *BuildingObjects* we refer to objects that exist within the building space (e.g., door, window, projector, heating, ventilation and air conditioning (HVAC) device) and may be used for realizing an action (e.g., in case of an object of type “window”, send a recommendation for closing the window). By *Sensor/Device* we refer to any sensor node able to provide data upon getting measurements for a specific parameter, denoted as *ObservedProperty* in the semantic model. Each *Sensor/Device* has a category type that in case of energy related sensors can be related with the monitoring of consumption, production or storage of energy.

Another basic concept that is introduced by IoT-Energy is the sensor data stream, denoted as *DataStream*. A *DataStream* generates *ObservationValues* for a specific *ObservedProperty* of a *Device/Sensor*. Different types of *DataStreams* may be activated for providing real-time or aggregated data.

For supporting the representation of data mining and analysis processes and the relations among the provided input and output data, concepts from the Linked Data Analytics Ontology (LDAO) are inherited [23]. Each *ObservationValue* is considered as an *AnalyticInputNode*. A set of *AnalyticInputNodes* are used as an *AnalyticInputCollection* for the realization of an *AnalyticProcess* and the production of *AnalyticResultNodes*.

#### 2.2.2. Behavioral Intervention Semantic Model

**Figure 5:**
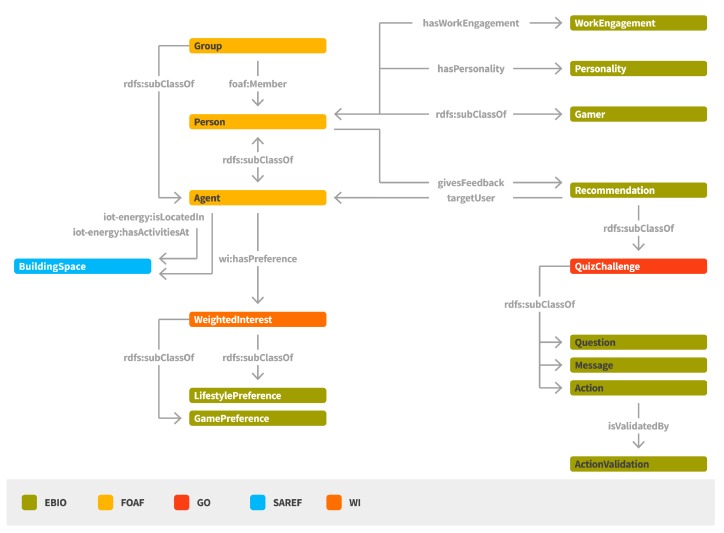
Behavioral intervention semantic model.

The Behavioral Intervention Semantic Model (EBIO) [21,22] aims to represent a set of concepts related to the behavioral profile of occupants in smart buildings and, thus, to facilitate the categorization of users in specific profiles and the provision of personalized content and recommendations for achieving behavioral change (Figure 5). The main concepts represented in EBIO regard the *Agent* and the *Recommendation*.

An *Agent* can be a *Person* or a *Group* where personalized recommendations can be sent. A *Person* has a “Personality” profile for denoting personality traits (e.g., extraversion, agreeableness, conscientiousness, emotional stability, openness to experiences). *WorkEngagement* characteristics can be also associated with a *Person*, mainly by providing indications with regards to the positive work-related state of fulfilment that is characterized by vigor, dedication, and absorption. A *Person* can be also be classified with regards to its gaming preferences, classification that can be proven very useful for providing the suitable content and application interaction mode (e.g., socializer, free spirit, achiever, disruptor). A *Person* may also have a set of interests denoted as *WeightedInterests* that may regard, among others, its game preferences (e.g., rewards, badges, points, levels) or lifestyle preferences.

Different types of *Recommendations* can be provided to *Persons* targeting at their behavioral change. Such *Recommendations* can have the form of a *Message*, a *QuizChallenge*, an *Action* or a *Question*. An *Action* is associated with an activity or a series of activities whose result contributes to elimination of a certain energy waste cause. A *Question* can be posed to a group of people, leading to the collection of crowd-sensing feedback (e.g., information regarding their comfort level). It should be noted that in the context of ENTROPY, the *Group* concept represents a group of users that share a common characteristic (e.g., tendency to sacrifice comfort for energy efficiency). A user can give a positive or negative *Feedback* to a *Recommendation* that is later utilized for the generation of new personalized recommendations.

### 2.3. Intelligent Energy Management and Awareness Services

Based on the semantically-mapped storage of the collected data in the ENTROPY big data repository, and through the definition of a set of REST APIs, various services can be designed and provided through the ENTROPY platform. Such services include the recommendation engine for providing personalized recommendations to end users, as well as the data mining and analysis mechanisms for providing behavioral and energy analytics. It should be noted that these mechanisms work in a complementary fashion, since produced output from an analysis process can trigger the provision of a new recommendation. Similarly, the feedback provided by end users based on the consumption of recommendations can lead to analysis and classification of end users in specific personality or gamer types.

#### 2.3.1. Recommendation Engine

**Figure 6:**
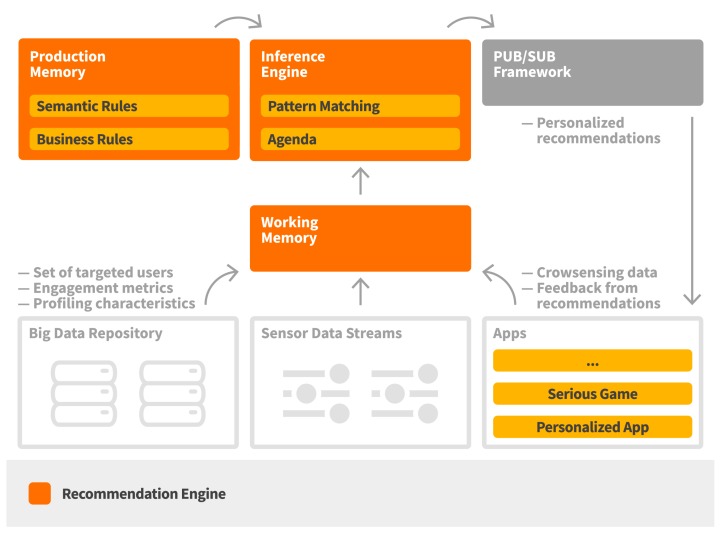
Recommendation engine workflow.

The recommendation engine is responsible for providing context-aware and personalized recommendations taking into account the occupants’ behavioral profiles. It is implemented based on Drools, a rules-based management system [25]. It consists of the working memory, where facts are introduced based on the provided data, the production memory, where the set of defined rules are available, and an inference engine that supports reasoning and conflict resolution, as well as triggering of the appropriate recommendations (see Figure 6).

Triggering of recommendations follows a continuous match-resolve-act approach. Specifically, the match phase regards the mapping of the set of applied rules which are satisfied based on the available data, the resolve phase regards the process of conflict resolution, if any, among the satisfied rules, while the act phase regards the triggering of the recommendations towards the group of the target users. Rules are mostly related with the identification of context change, especially with regards to the location of the end users or changes in the observed values in the activated data streams.

A rule consists of a condition element and a recommendation template in the action part, which connects a context change with specific target user group criteria. When a rule is fired due to a context change (e.g., when average CO_2_ measurement within an hour exceeds the defined threshold), the recommendation engine selects the set of target users based on the defined user attribute filters (e.g., players who have activities at a certain location, users that are classified as highly responsive at the proposed actions through the personalized recommendations, users that satisfy specific behavioral criteria) and creates a personalized recommendation for each of them by using the defined recommendation template. Following this, the set of recommendations are published in a publish/subscribe framework and made available for consumption by the set of personalized applications and serious games.

**Figure 7:**
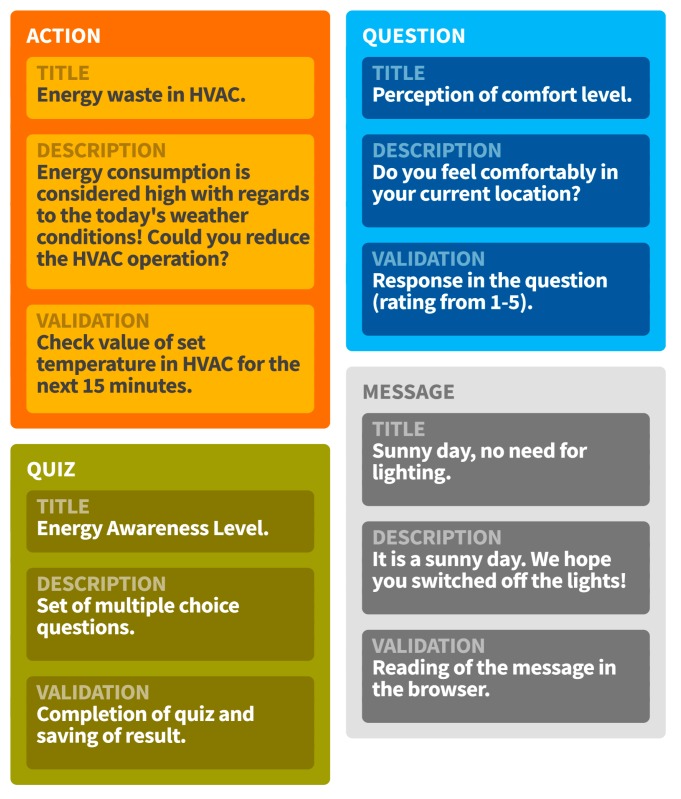
Indicative recommendations.

A produced recommendation contains the target user, the related content, the measurement attributes that are involved in the creation of the recommendation, the possible reward for the completion of the recommendation, as well as the validation method for it. The attributes involved in the creation of the recommendation is provided for gamification purposes, since different measurements may work towards earning different rewards (e.g., the points earned from completing a task regarding CO_2_ may have an impact on earning a so called “Refresher Badge”). The rewards are registered to a user upon the completion and validation of a recommendation, which differs per type of recommendation. For instance, an action may be validated by checking the status of the sensors on the involved building objects (e.g., a window), while the validation of a quiz is done inherently by answering all the questions. A set of indicative recommendations are provided in Figure 7.

It should be noted that reasoning mechanisms are applied based on the designed ENTROPY semantic models. An implementation of a reasoning business logic is realized based on Drools, mainly for exploiting the high performance and scalability characteristics of Drools-based systems, compared to ontology-based reasoning [26], as well as the separation of the rules-definition business logic from the core ENTROPY functionalities. Campaign managers are able to define and update or extend the set of rules applied for inference purposes in a dynamic way, compared to static approaches realized in ontology-based reasoning, while rules declaration can be realized in a non-technical way in order to be understandable to people that are not domain experts [27]. In this way, both performance and rules definition complexity aspects are tackled.

However, given that an OWL implementation offers advantages in terms of rules expressiveness as well as exploitation of the semantic properties of a semantic model, the development of a module is realized, denoting set of rules in Drools native rule language (DRL) able to support the set of semantic properties. It should be noted that the examination of such a solution was also suggested by existing performance evaluation studies comparing Drools and ontology-based reasoning [28]. In more detail, dynamic production of new knowledge is supported through the execution of specific rules that are responsible to produce reasoning on the fly. These rules are able to perform class transitiveness (i.e., if a base-class belongs to a class that has also a parental class, then the new knowledge is the fact that the base-class belongs also to the parental class), supertype inheritance (i.e., if there is an instance of class that has a parental class then the new knowledge is the fact that a replica of the same instance instantiates the parental class), property transitiveness and sub-property transitiveness. The inference business logic is implemented using the first-order logic capabilities of the Drools engine instead of relying on third-party reasoners, enhancing a significant amount of the overall system efficiency.

Drools-based reasoning mechanisms are also considering a set of axioms that are denoted throughout the usage of the platform for declaring advanced relationships that are observed. For instance, given that by the interpretation of a first set of questionnaires results, it is denoted that humanitarian-oriented persons show a preference for badges and roles in the used mobile applications, we can define a class axiom in OWL Manchester syntax such as “(hasPreference value Badge and hasPreference value Role) subClassOf Humanitarian”. Such an axiom is included in the real-time reasoning process, leading to production of the relevant knowledge each time the axiom description is validated.

#### 2.3.2. Data Mining and Analysis Services

Another service that is implemented and provided within the ENTROPY platform regards the support of a set of big data mining and analysis techniques towards the extraction of energy and behavioral analytics. Insights provided with regards to the energy usage in smart buildings, as well as the behavioral characteristics of the occupants, may lead on one hand on increase of their energy awareness and on the other hand on targeted recommendations for reducing energy consumption.

The supported set of analytics processes concerns descriptive, predictive, classification, clustering, and prescriptive analytics [5,29]. Descriptive analytics are providing summary information regarding the energy usage, as well as other environmental or behavioral attributes. Predictive analytics are providing estimates for usage of energy the upcoming period, as well as examining the relationship among energy consumption and set of parameters, such as average temperature, heating or cooling degree days, day of the week, etc. The considered algorithms include linear regression, multiple linear regression, support vector regression, and principal component analysis. Classification and clustering analytics are applied for identifying or classifying collective behaviors among the involved users. Based on the identification of groups, targeted interventions may be planned, while the produced groups may be also considered as input towards a group-aware forecasting analysis. The considered algorithms include artificial neural networks, Bayesian regularized neural networks, random forest, k-means, density-based spatial clustering, and hierarchical clustering. Prescriptive analytics are applied for combining analytics results with automation solutions considering the interplay among energy efficiency and comfort level of occupants.

**Figure 8:**
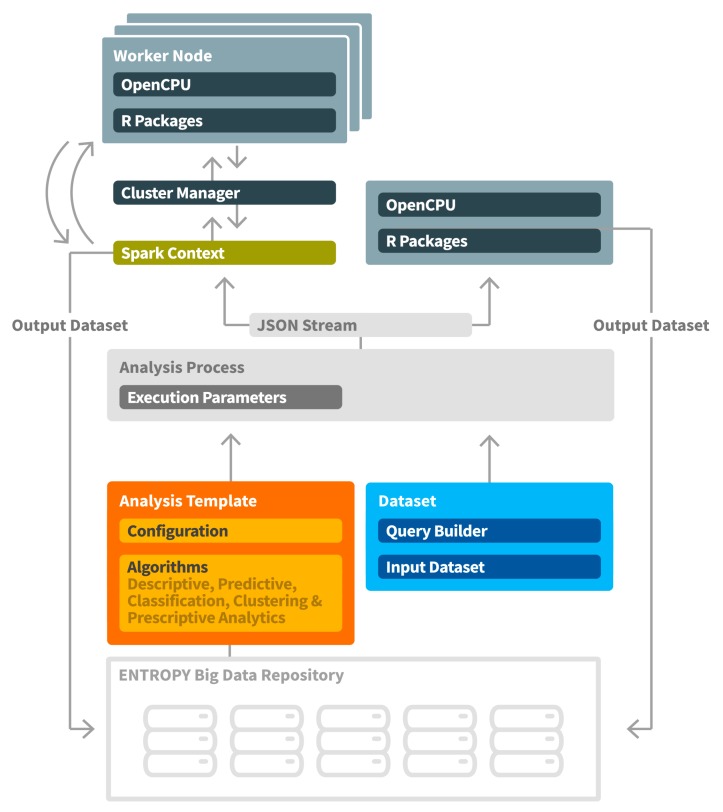
Data mining and analysis workflow.

**Figure 9:**
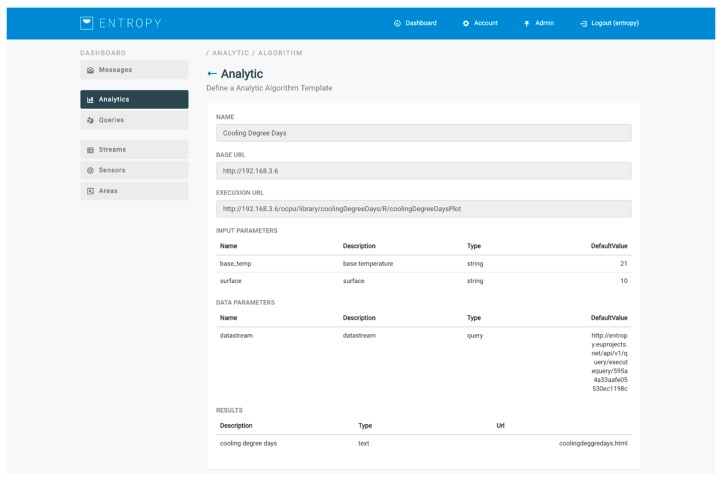
Indicative algorithm analysis template.

The workflow followed for the support of data mining and analysis techniques is depicted in Figure 8. An analysis process is based on the selection of an analysis template and the selection of the queries to be executed for providing the input datasets (training and/or evaluation datasets). Each analysis template represents a specific algorithm and provides to the user the flexibility to adjust the relevant configuration parameters. Such parameters include input parameters for the algorithm along with their description and their default value, as well as output parameters along with their type (text, image, data, html). An indicative analysis template for the calculation of heating or cooling degree days per day [30] for a monthly period is depicted in Figure 9. A set of analysis templates can be made available and be used for initiating an analysis. It should be noted that an analysis process is also associated with a set of execution parameters that denote whether an analysis should be realized in a manual or automated way, as well as the periodicity factor for the latter case.

**Figure 10:**
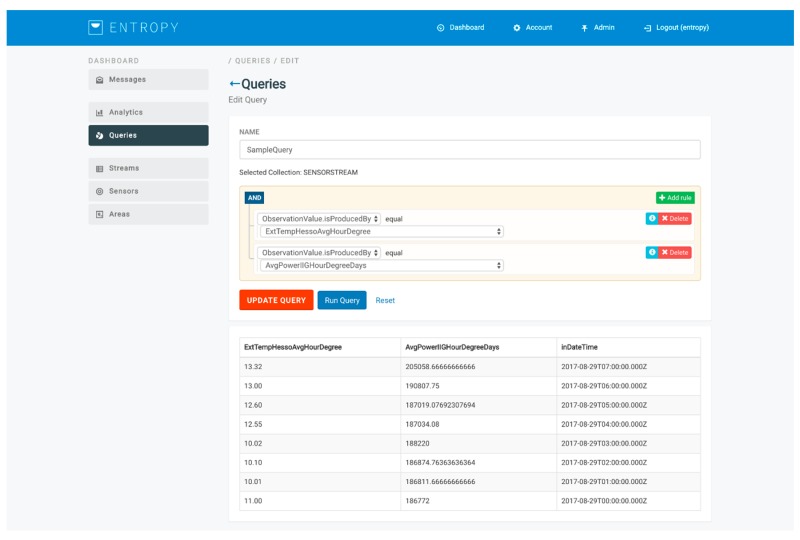
Indicative query design through the query builder.

The design of queries for obtaining the input datasets for the analysis is based on the development of a query builder over MongoDB, facilitating end users to easily prepare their input datasets. Two categories of queries are supported, namely queries for fetching data collected by sensor data streams (e.g., energy consumption, humidity, and indoor temperature data per hour for a specific room) and queries for fetching data related to the set of users participating at the energy efficiency campaign (e.g., a set of users with an educational level relevant to a Master’s degree). An indicative query for getting the average power consumption and the external temperature per hour is depicted in Figure 10. Upon the execution of the queries, streams of the input training or evaluation datasets are provided to the analysis toolkits.

In ENTROPY, the R Project for Statistical Computing [31], and the Apache Spark fast and general engine for large-scale data processing [32] are used for this purpose. Depending on the analysis needs in terms of big data management and performance aspects, the optimal tool per case may be selected. Interconnection of the ENTROPY components with the analysis toolkits is based on the OpenCPU system for embedded scientific computing that provides a reliable and interoperable HTTP API for data analysis based on R. In the case of large-scale data processing and the need for a big data analysis framework, the Apache Spark engine is used, where the analysis process is realized in a set of worker nodes, each one of which is hosting an Apache Spark OpenCPU Executor [33]. The set of worker nodes are formulating a cluster orchestrated by a cluster manager.

Upon the realization of an analysis, the produced results (output dataset) are also made available through a set of URLs providing access to the set of results, as defined in the output parameters of the analysis template. It should be noted that analysis results are also semantically mapped to the ENTROPY semantic models, based on the adoption of the LDAO ontology, as mentioned in the previous section.

At the current phase, a set of initial algorithms are considered, however, the overall implementation facilitates the incremental addition of further analysis mechanisms.

#### 2.3.3. Personalized Applications and Serious Games Development

**Figure 11:**
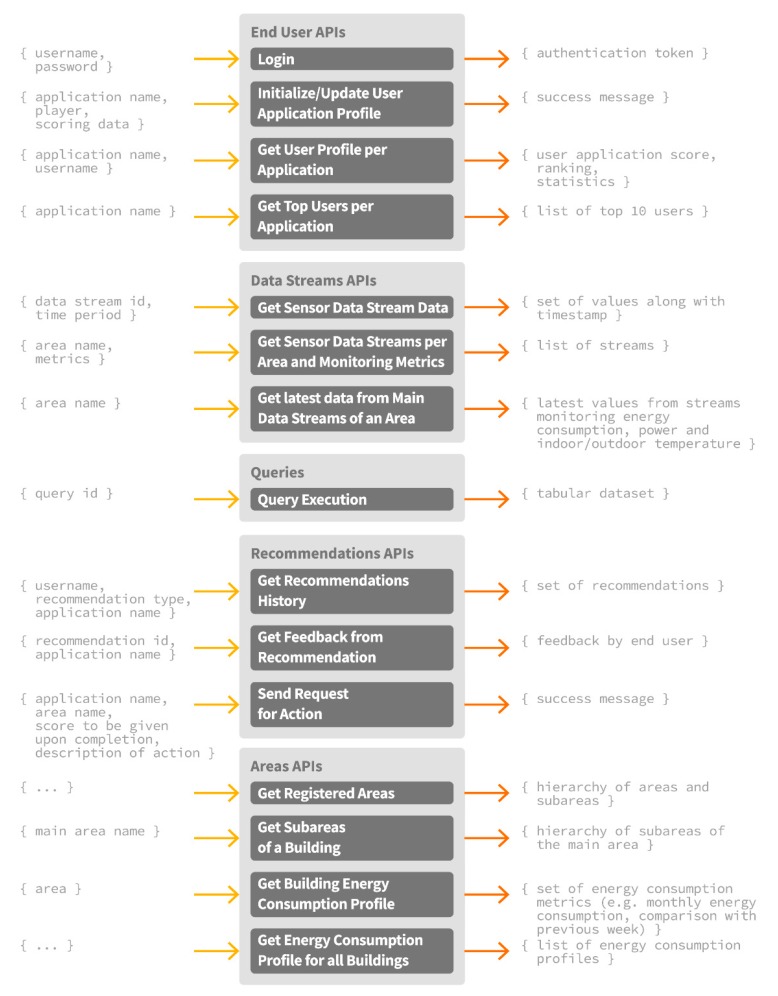
ENTROPY API overview.

In addition to the set of intelligent energy management and awareness services supported by the ENTROPY IT ecosystem, the development of mobile applications is facilitated. Given the unified representation of data through the semantic models independently of the underlying sensor infrastructure, as well as the design and implementation of set of REST APIs for accessing and storing data to the big data repository, personalized applications and serious games development is enabled, while their applicability may regard various smart building cases. Such APIs include, among others, the provision of information for the available building spaces and their energy consumption profiles, the activated sensor data streams, latest data per sensor data stream, the recommendations provided per user along with the collected feedback, the set of actions that may be requested to be realized by an end user, the execution of queries, user demographic data, functionalities for user registration, authentication and login in the IT ecosystem, initialization and update of the user profile per application, as well as the retrieval of the top users per application. An overview of the developed APIs along with the associated input and output parameters is provided at Figure 11. In each case, the set of GET or POST body parameters, the headers and the type of the response are specified, as detailed in [34]. The aforementioned functionalities can be proven beneficial towards the development of smart applications that can combine energy and behavioral data, along with the services provided by the ENTROPY IT ecosystem, and lead to the improvement of energy efficiency in smart buildings.

## 3. Improving Energy Efficiency at the University of Murcia

One of the pilot cases where the ENTROPY IT ecosystem is instantiated is at the University of Murcia in Spain. The pilot regards an energy efficiency campaign at three cases, namely the Faculty of Chemistry and two multi-disciplinary research centers. In each of these cases, a set of infrastructure sensors and actuators have been installed to capture energy-related, as well as environmental data. The three cases comprise of 25 energy smart meters and 190 HVACs monitoring energy consumption and temperature in various building spaces, ranging from a room or corridor level to the overall building.

The targeted users regard students, professors and the administrative staff. While students’ behavioral changes are tackled on the shared labs as a group, every professor has their own office, and so personal actions might be carried out. Contrary to the other two groups, the administrative staff is required to register when they enter or leave their offices by means of personal smart cards providing in this manner data regarding their presence.

Following some preliminary results of the aforementioned campaign for the building of the Faculty of Chemistry are detailed. These results regard the realization of a set of data mining and analysis processes with a twofold objective. On one hand, targeting at the prediction of the energy consumption for the following day and on the other hand targeting at the grouping of the set of considered building spaces based on the usage of the HVAC equipment during the day.

**Figure 12:**
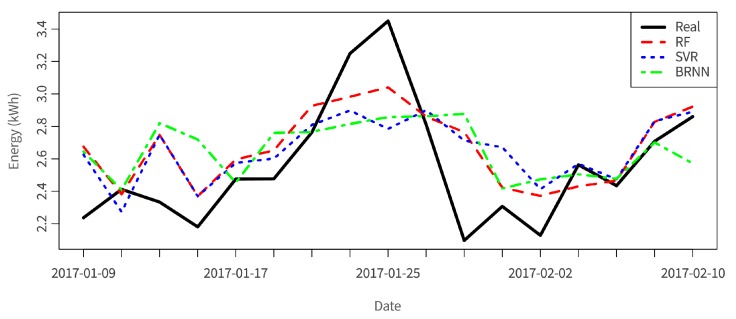
Analysis results: energy consumption prediction based on environmental variables.

The first part of the analysis is realized per day at evening time and is based on weather forecasting data provided by the Weather Underground API service [35] and energy consumption data of the overall building. The output data regards energy consumption for the following day. The applied algorithms are random forest (RF), support vector regression (SVR), and Bayesian regularized neural network (BRNN), providing the root-mean-square error (RMSE) of the prediction, which varies between 0.4 and 0.6 for the tested algorithms, where $RMSE=\sqrt{\left(\frac{\sum {\left(y_{i}-\bar{y_{i}}\right)}^{2}}{n}\right)}$. In Figure 12, the application of the built models to the test dataset is shown for a set of dates, where random forest provides the best results. When normalizing the RMSE by the mean of the real tested values, we obtain the coefficient of variation (CVRMSE). CVRMSE is used to avoid ambiguity when comparing models. In this case it varies between 9.5% (RF) and 12.7% (BRNN), meaning good predictive results. By having accurate predictions regarding the energy consumption, optimal planning of usage of energy can be achieved.

**Figure 13:**
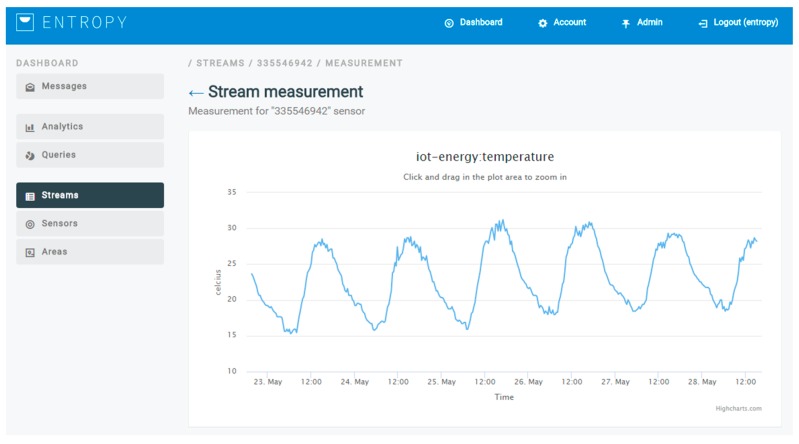
Outdoor temperature at the University of Murcia Campus.

**Figure 14:**
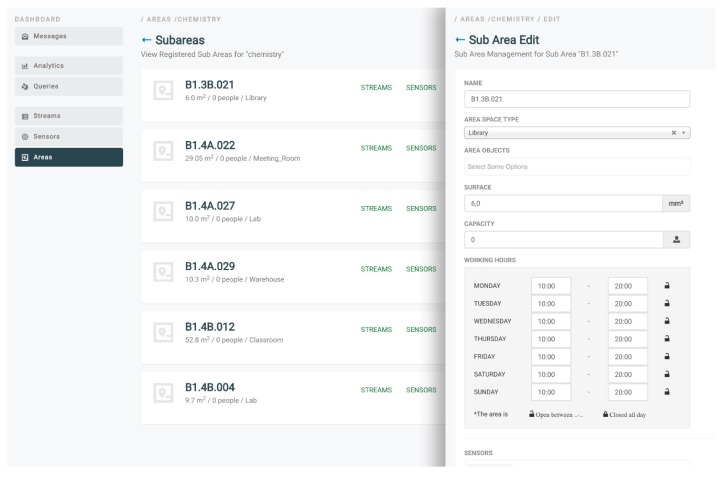
Building space and subspaces registration at the University of Murcia Campus.

In the second part of the analysis, information regarding the usage of the HVACs in a set of building spaces is collected and used for clustering purposes. Based on the cluster where a building space is assigned, targeted recommendations to the users that have activities in this building space may be provided. The information used for clustering purposes regards the state of the HVAC devices (on/off), the indoor and outdoor temperature, as well as the target temperature set. An indicative graph of the outdoor temperature for a weekly period is depicted in Figure 13, as it is produced by the ENTROPY platform, while an indicative figure of the configuration provided for a registration of building spaces and subspaces is also depicted in Figure 14.

**Figure 15:**
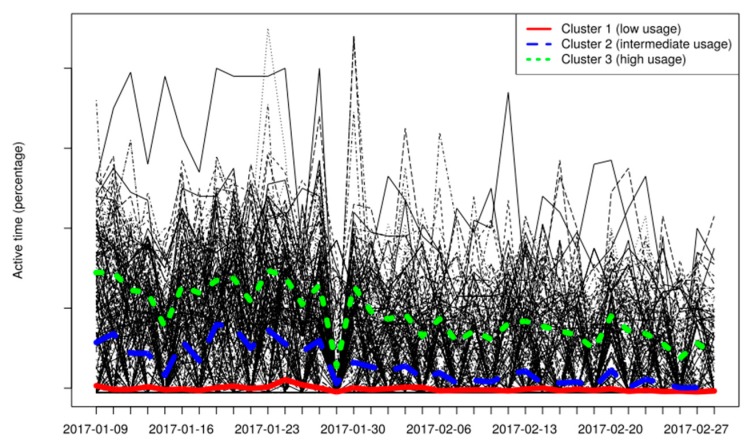
Analysis results: Clustering of building spaces according to their HVAC usage through time (Cluster 1/2/3: red/blue/green line denoting trajectory of set of building spaces with low/intermediate/high usage patterns, accordingly).

The set of algorithms applied for clustering purposes are hierarchical clustering, longitudinal k-means and density-based spatial clustering of applications with noise. In Figure 15, the three trajectories of the HVAC groups that hierarchical clustering identifies for working days of January and February 2017 are colored. Each black line corresponds to the usage graph (percentage of daily active time period) of a single HVAC device through both months. Clusters 1, 2, and 3 regard building spaces with low, intermediate, and high usage patterns, accordingly. Each cluster trajectory line corresponds to the mean daily value of the set of building spaces that belong to the cluster. These clusters can be introduced in the energy consumption model as input variables, since the energy consumption is linearly dependent to the HVAC usage.

**Figure 16:**
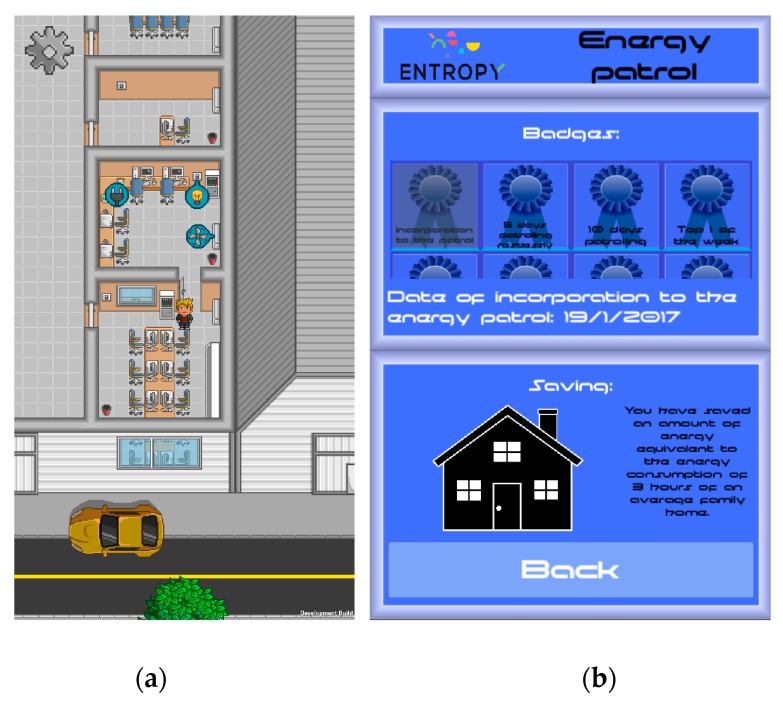
Screenshots from interactive games: (**a**) My Green Avatar; and (**b**) The Energy Patrol.

For interacting with end users participating in the energy efficiency campaign and providing personalized recommendations, two games have been developed by exploiting the ENTROPY IT ecosystem. Indicative screenshots from these games are depicted in Figure 16. The first game (Figure 16a)—called “My Green Avatar”—allows users within the same building to report their actions to achieve energy savings (e.g., switch-off HVACs, turn off appliances or lights, and the like) by means of their own virtual avatar. Then, such actions are confirmed by analyzing the data streams of the related actuators. The second game (Figure 16b)—called “The Energy Patrol”—provides recommendations to users to undertake specific actions to improve the energy savings of their building like turning off the lights in potentially empty rooms or adjust the temperature of the HVACs to an efficient setting. Such recommendations are provided to end users based on a set of rules defined in the ENTROPY recommendation engine and are consumed in real-time through the deployed publish/subscribe framework. Validation of the successful realization of the proposed actions is also supported, exploiting the mechanisms provided by the recommendation engine.

## 4. Conclusions

In the current article, a novel IT ecosystem is presented that aims to improve energy efficiency in smart buildings through behavioral change of the occupants, based on the exploitation of emerging ICT technologies.

A set of innovative characteristics of the ENTROPY IT ecosystem are detailed. The set of open and extensible mechanisms for sensors registration, configuration, and sensor data management at the edge or the core part of the infrastructure facilitates the easy adoption of the overall solution and its instantiation in diverse and heterogeneous infrastructure cases. The representation of data based on the specification of energy and behavioral semantic models facilitate the unified access to them by numerous applications and services as well as their interconnection with available open and linked data for further processing. The set of data analysis and recommendation services, designed in a way that they collaborate among each other can lead to targeted recommendations, energy and behavioral analytics, actions and decisions with direct impact on behavioral change of occupants and, thus, energy efficiency increase. Finally, the set of REST APIs provided for consuming the set of available services can boost the design and development of personalized applications and serious games targeted to specific type of buildings with minimal effort.

The detailed IT ecosystem can be applied in diverse cases with minimal configuration effort, the supported energy management and awareness services can be easily consumed while the design and development of further services and mobile applications is highly facilitated through the exploitation of the unified way of representation of the collected data.

Building upon the presented energy-aware IT ecosystem and taking into account the set of initial results produced, several open issues and ideas for extensions are identified. Based on the existing implementation of data mining and analysis services, the design and implementation of a set of data mining and analysis processes stemming from various tools can be realized, leading to advance insights through the processing of energy data. Such tools include the R statistics project, SparkR as a light-weight frontend to use Apache Spark from R, as well as analysis software implemented via other tools (e.g., Python scripts), exploiting the interfaces provided through OpenCPU. With regards to the recommendation engine, extensive performance evaluation results for the usage of Drools for semantic reasoning purposes taking into account the number of introduced rules and the volume of the processed data can be realized, leading to meaningful and exploitable insights for adopting such a solution in energy management solutions as well as other domains. In parallel, collection of feedback for the provided semantic models can lead to extensions or minor modifications of them aiming to serve a wider community and improve data interoperability aspects. Further extensions in the FIWARE enablers can be also implemented and proven beneficial in order to introduce advanced complex event processing rules for improving data quality prior to transmitting and processing them at the centralized infrastructure. Finally, realization of a set of energy efficiency campaigns, part of which are already planned to be realized within the ENTROPY H2020 project, and evaluation of the potential for reducing energy consumption through the usage of the ENTROPY IT ecosystem services has to be achieved combined with a set of dissemination activities for adoption of the ecosystem by a wider community.

## Supplementary Materials

The following are available online at http://www.mdpi.com/1424-8220/17/9/2054/s1; Video S1. Introduction to ENTROPY; Video S2. Walkthrough in the ENTROPY platform.
